# Antinociceptive Activity of Chemical Components of Essential Oils That Involves Docking Studies: A Review

**DOI:** 10.3389/fphar.2020.00777

**Published:** 2020-05-29

**Authors:** Davidson Barbosa Assis, Humberto de Carvalho Aragão Neto, Diogo Vilar da Fonsêca, Humberto Hugo Nunes de Andrade, Renan Marinho Braga, Nader Badr, Mayara dos Santos Maia, Ricardo Dias Castro, Luciana Scotti, Marcus Tullius Scotti, Reinaldo Nóbrega de Almeida

**Affiliations:** ^1^Psychopharmacology Laboratory, Institute of Drugs and Medicines Research, Federal University of Paraíba, João Pessoa, Brazil; ^2^First Faculty of Medicine, Charles University, Prague, Czechia; ^3^Cheminformatics Laboratory, Institute of Drugs and Medicines Research, Federal University of Paraíba, João Pessoa, Brazil

**Keywords:** nociception, essential oils, docking, *in silico*, molecular target

## Abstract

**Introduction:**

Pain is considered an unpleasant sensory and emotional experience, being considered as one of the most important causes of human suffering. Computational chemistry associated with bioinformatics has stood out in the process of developing new drugs, through natural products, to manage this condition.

**Objective:**

To analyze, through literature data, recent molecular coupling studies on the antinociceptive activity of essential oils and monoterpenes.

**Data source:**

Systematic search of the literature considering the years of publications between 2005 and December 2019, in the electronic databases PubMed and *Science Direct*.

**Eligibility Criteria:**

Were considered as criteria of 1) Biological activity: non-clinical effects of an OE and/or monoterpenes on antinociceptive activity based on animal models and *in silico* analysis, 2) studies with plant material: chemically characterized essential oils and/or their constituents isolated, 3) clinical and non-clinical studies with *in silico* analysis to assess antinociceptive activity, 4) articles published in English. Exclusion criteria were literature review, report or case series, meta-analysis, theses, dissertations, and book chapter.

**Results:**

Of 16,006 articles, 16 articles fulfilled all the criteria. All selected studies were non-clinical. The most prominent plant families used were Asteraceae, Euphorbiaceae, Verbenaceae, Lamiaceae, and Lauraceae. Among the phytochemicals studied were α-Terpineol, 3-(5-substituted-1,3,4-oxadiazol-2-yl)-N′-[2-oxo-1,2-dihydro-3H-indol-3-ylidene] propane hydrazide, β-cyclodextrin complexed with citronellal, (−)-α-bisabolol, β-cyclodextrin complexed with farnesol, and p-Cymene. The softwares used for docking studies were Molegro Virtual Docker, Sybyl^®^X, Vlife MDS, AutoDock Vina, Hex Protein Docking, and AutoDock 4.2 in PyRx 0.9. The molecular targets/complexes used were Nitric Oxide Synthase, COX-2, GluR2-S1S2, TRPV1, β-CD complex, CaV_1_, CaV_2.1_, CaV_2.2_, and CaV_2.3_, 5-HT receptor, delta receptor, kappa receptor, and MU (μ) receptor, alpha adrenergic, opioid, and serotonergic receptors, muscarinic receptors and GABA_A_ opioid and serotonin receptors, 5-HT_3_ and M_2_ receptors. Many of the covered studies used molecular coupling to investigate the mechanism of action of various compounds, as well as molecular dynamics to investigate the stability of protein-ligand complexes.

**Conclusions:**

The studies revealed that through the advancement of more robust computational techniques that complement the experimental studies, they may allow some notes on the identification of a new candidate molecule for therapeutic use.

## Introduction

Pain was conceptualized for the first time in 1986 by the International Association for the Study of Pain (IASP); being defined as an response of the Central Nervous System to a tissue injury or an emotional change and classifiable as acute or chronic (McKune et al., 2015), adaptive or non-adaptive (Hellyer et al., 2007; de Oliveira Alves et al., 2017), and as physiological or pathological (Klaumann et al., 2008). The neural process of coding and processing noxious stimuli is called nociception (Naidu and Pham, 2015).

Various animal models have been developed with the purpose of understanding the mechanisms involved in nociception, including hot-plate, Hargreaves, von Frey, Randall-Selitto, capsaicin, glutamate, and formalin methods among others. Most of the non-clinical experimental models involve the use of stimuli; of a chemical, thermal, or mechanical nature where characteristic behaviors reflecting the nociceptive response are recorded. Animal models should be able to predict the effects of new drugs in humans, as well as clinical analgesic sensitivity. For this purpose, the animals are usually rodents (mice and rats), but alternative species have been used (Barrot, 2012; Barrett, 2015).

Computational chemistry associated with bioinformatics has led the process of developing new drugs with various activities, especially analgesia. Molecular docking is a computational technique that predicts the positioning (orientation and conformation) of a ligand (drug or molecule of therapeutic interest) in a target interaction site, and aids in the understanding of biological activity, by both explaining and predicting possible interactions, and helping to evaluate pharmacological properties and relations between chemical structure and biological activity (Chen and Zhi, 2001; Kirchmair et al., 2012). Thus, for molecules of therapeutic interest, molecular docking serves as a predictive model that can contribute to *in vivo* evaluations of pharmacological activity.

Since natural products derived from plants have a wide variety of bioactive chemical compounds, they present an important alternative in the search for therapeutic agents. Essential oils and their constituents, monoterpenes and sesquiterpenes, have several pharmacological properties, among them the potential analgesic effect. (Bahmani et al., 2014; Gomathi and Manian, 2015; de Oliveira Júnior et al., 2018). However, these products are often unexplored (Leonardão et al., 2016), and this fact collaborates to promote the scientific hypothesis related to the antinociceptive effect, including performing the molecular anchorage studies.

The objective of this study was to conduct a survey of recent molecular docking studies involving the antinociceptive activity of essential oils and monoterpenes.

## Materials and Methods

### The Question Under Study

This systematic review was carried out to address the specific question: “What are the scientific findings associating non-clinical animal studies and *in silico* analysis when evaluating the antinociceptive activity of essential oils?”

### Search Strategy and Selection of Studies

The guidelines of the PRISMA guide of Systematic Reviews and Meta-Analyses) (Liberati et al., 2009) were followed. Two databases were systematically searched for experimental antinociceptive *in vivo* studies and *in silico* analysis of essential oil activity—as published through December 20, 2019 (**Table 1**).

**Table 1:** Search mechanism and bibliographic databases used to choose the articles for this review.

Primary bibliographic sources	Search strategy (descriptors/combinations with Boolean operators)
**Science Direct** (2005**–**2019)	(essential oils) AND (monoterpenes OR sesquiterpenes) AND (antinociceptive) (essential oils) AND (monoterpenes OR sesquiterpenes) AND (antinociceptive) AND (docking) (essential oils) AND (monoterpenes OR sesquiterpenes) AND (*in silico*) (essential oils) AND (monoterpenes OR sesquiterpenes) AND (antinociceptive) AND (*in silico*) (essential oils) AND (monoterpenes) OR (sesquiterpenes) AND (pain) AND (*in silico*) (essential oils) AND (monoterpenes) OR (sesquiterpenes) AND (pain) AND (docking)
**Pubmed** (2005–2019)	(essential oils) AND (monoterpenes OR sesquiterpenes) AND (antinociceptive) (essential oils) AND (monoterpenes OR sesquiterpenes) AND (antinociceptive) AND (docking) (essential oils) AND (monoterpenes OR sesquiterpenes) AND (*in silico*) (essential oils) AND (monoterpenes OR sesquiterpenes) AND (antinociceptive) AND (*in silico*) (essential oils) AND (monoterpenes) OR (sesquiterpenes) AND (pain) AND (*in silico*) (essential oils) AND monoterpenes) OR sesquiterpenes) AND (pain) AND (docking)

### Eligibility Criteria

Systematic screening of the articles was performed by two independent examiners according to the following inclusion criteria:

1. Biological activity: non-clinical effect of essential oil (EO) on nociceptive activity based on animal models and *in silico* analysis.
  - Primary outcomes of interest: acetic acid-induced abdominal writhing, formalin-induced nociception, orofacial formalin-induced nociception test, chronic muscle pain test, tail-flick test, hot plate test, tail immersion test, and von Frey test.
  - Secondary outcomes of interest—Studies of the antinociceptive mechanisms of action: Involvement of opioid receptors, Involvement of ATP-sensitive K_ATP_channels, *in silico* (molecular docking) analyses.
2. Plant material and chemical elucidation: chemically characterized essential oils and/or their isolated constituents from aromatic plants.
3. Study design: non-clinical animal studies, clinical studies, and *in silico* analysis to evaluate the antinociceptive activity of essential oils.
4. Methodological quality: accuracy of methods and outcomes; internal and external validity.
5. Language: for articles written in English, in cases of inconsistency, the examiners would give the final verdict on which articles should be included in this review would be reached by consensus.

### Study Selection

To compose the sample of this review, a database was initially searched according to the strategies mentioned in **Table 1**. In this phase of the search, the results were compared, and duplicated articles found between the databases were excluded, and studies that were explicitly different from the criteria and objective of this review were excluded through the evaluation of titles and abstracts. Thus, 16 articles were included in this review, which deals with evaluations of monoterpene and sesquiterpene antinociceptive activity *via in silico* docking studies from 2011 to December 2019.

### Data Collection and Analysis

The following variables were collected: plant family, plant species, source, phytochemical, molecular target, route of administration, animal species, antinociceptive test, software, results, country, and reference.

This information is detailed in **Tables 2** and **3**. The research data were analyzed based on the ARRIVE guidelines (Animal Research: Reporting of *In Vivo* Experiments) published by the Animal Center for the Replacement, Refinement & Reduction of Animals in Research (Kilkenny et al., 2010).

**Table 2:** Information ethnobotanical, molecular, pharmacological, and docking programs used *in vivo* studies involving the antinociceptive activity of essential oils.

Plant Family	Plant Species	Phytochemical	Molecular target	Source	Route of administration	Animal(s) species	Antinociceptive test	Software	Results	Country	Reference
*Verbenaceae*	*Lippia grata Schauer*	*Bicyclogermacrene*	Alpha Adrenergic, Opioid and serotoninergic receptors.	Northeastern Brazil	Gavage	Swiss	Chronic muscle pain model	Molegro Virtual Docker v. 6.0.1.	Anti-hyperalgesic activity of LG-β-CD seems to involve opioid and serotoninergic receptors	Brazil	Siqueira-Lima et al., 2017.
*Lauraceae*	*Cinnamomum* *sintoc bl*.	*Eugenol*	COX-2.	Yogyakarta district	Ip	Swiss albino mice	Acetic acid induced writhing method	–	Eugeunol presented better molecular prediction for naproxen (control ligand-blue carbon) at binding site of COX-2.	Indonesia	Sumiwi et al., 2015.
Euphorbiaceae	*Croton* *conduplicatus* Kunth	Spathulenol Caryophyllene oxide	Muscarinic receptors and GABAA.	Northeastern Brazil	Ip	Male Swiss mice	Acetic-acid-writhing-induced nociception, Formalin-induced nociception, Hot plate test.	Molegro Virtual Docker, v. 6.0.1.	Majority EO compounds (1,8-cineole, spathulenol, caryophyllene oxide and p-cymene) were dose-dependent and appear to involve muscarinic, opioid, and GABAA receptors.	Brazil	de Oliveira Júnior et al., 2018.
*Euphorbiacea*e	*Croton* *conduplicatus Kunth*	*(E)-caryophyllene* *Caryophyllene oxide*	Muscarinic receptors.	Northeastern Brazil	Ip	Male Swiss mice	Acetic-acid-writhing-induced nociception, Formalin-induced nociception, Hot plate test.	Molegro Virtual Docker.	Majority compound structures of camphor, caryophyllene oxide, and (E)-caryophyllene submitted to molecular docking—EO acts through central and peripheral mechanisms, possibly involving K_ATP_ channels and muscarinic receptors	Brazil	De Oliveira Junior et al., 2017.
Lamiaceae	*Hyptis pectinata*	*Caryophyllene oxide* *Germacrene D*	Opioid and serotonin receptors.	Malhada dos Bois (Sergipe State), in northeastern Brazil	Subcutaneous	Male Swiss mice	Acid Saline-Induced Chronic Muscle Pain, Mechanical Sensitivity of the Muscle (Primary Hyperalgesia), Mechanical Sensitivity of the Paw (secondary hyperalgesia)	Molegro Virtual Docker v. 6.0.1.	Main components of EOH were β- caryophyllene, caryophyllene oxide, germacrene D, and linalool. Central analgesic activity seems to be evoked by the action of NE-EOH on the opioid and serotonin systems.	Brazil	Quintans-Junior et al., 2018.
Asteraceae	*Ageratina glabrata*	Chromene derivative Meloxicam	COX-2.	Mexico	Ip	Sprague Dawley rats	Hot plate	Sybyl^®^X software suite.	Antinociceptive effect (COX-2 inhibition) Not affected by hormonal changes	Mexico	García et al. (2011)
Asteraceae	*Achillea falcata L*.	*trans*-Sabinol	–	Syria	Ip	BALB/c mice	Ach writhing Hot plate Tail immersion	–	Antinociceptive effect Toxicity of some derivatives not ruled out	Serbia	Radulović et al. (2015)
Asteraceae	*Vanillosmopsis arborea Baker*	*(*−*)-α-bisabolol*	5-HT3 and M2 receptors.	Brazil	Gavage	Swiss mice Wistar rats	Formalin Capsaicin Acidic saline Glutamate	Molegro Virtual Docker.	Antinociceptive effect	Brazil	Leite et al. (2019)

This table summarizes the main results obtained in research where essential oils from natural products were used to test for possible antinociceptive activity.

**Table 3:** Information ethnobotanical, phytochemical, molecular, pharmacological, and docking programs used *in silico* studies involving the antinociceptive activity isolated from essential oils.

Phytochemical	Molecular target	Chemical marker	Route of administration	Animal(s) species	Antinociceptive test	Software	Results	Country	Ref
α-Terpineol	Nitric Oxide Synthase enzyme	TP, amino guanidine, dexamethasone, Nitro- L-arginine methyl ester (L-NAME)	Subcutaneous	Male Swiss	Mechanical hyperalgesia was assessed by means of digital von Frey	Molegro Virtual Docker v. 6.0.1.	Antinociceptive effect of TP probably occurs *via* mechanisms related to modulation of oxidative stress, with maintenance of endogenous antioxidant substances and reduction of iNOS levels.	Brazil	Gouveia et al., 2018.
3-(5-substituted-1,3,4-oxadiazol-2-yl)-*N*′-[2-oxo-1,2-dihydro-3*H*-indol-3-ylidene]propane hydrazides	COX-2	indomethacin	Po	Albino Wistar mice	Hot plate	Vlife MDS.	Antinociceptive effect	India	Kerzare et al., 2016
β-cyclodextrin (CT-βCD) complexed with citronellal (CT)	GluR2-S1S2	1FTJ protein complexed with glutamate	Po	Swiss mice	Digital von Frey Grip strength meter	AutoDock Vina.	CT-βCD has a greater analgesic effect than the free form (CT alone)	Brazil	Santos et al., 2016
(−)-α-bisabolol	TRPV1	–	Intraocular	Swiss mice	Hypertonic saline-induced corneal nociception	Hex Protein Docking (HEX)	Nanoencapsulated BISA is topically active—attenuates 5 M NaCl-induced corneal nociception	Brazil	Teixeira et al., 2017
(−)-α-bisabolol	TRPV1	–	Po and topical	Adult male Swiss albino mice and adult male Wistar rats	Orofacial formalin test Orofacial cinnamaldehyde test Temporomandibular joint formalin test	Hex Protein Docking (HEX)	The study confirmed the anti-nociceptive effect of BISA on orofacial pain. The effect may in part be due to TRPA1 antagonism	Brazil	Melo et al., 2017
B-cyclodextrin complexed with farnesol	β-CD complex	–	Ip	Male Swiss mice	Formalin, Orofacial capsaicin, glutamate	AutoDock 4.2 software in the PyRx 0.9.	Farnesol complexed with β-CD presented best antinociceptive activity, probably *via* 5-HT3 receptor	Brazil	(Silva et al., 2017).
p-Cymene	CaV1, CaV2.1, CaV2.2 and CaV2.3	p-cymene, nicardipine, ω-agatoxin IVA, ω-conotoxin GVIA, and N-Triazole Oxindole	Subcutaneous	Male Albino Wistar mice	Digital von Frey Grip strength meter	Molegro Virtual Docker	p-Cymene was able to reduce calcium current density	Brazil	Santos et al., 2019
α-terpineol	5-HT receptor Delta receptor Kappa receptor MU receptor	–	Ip	Male Swiss mice	Mechanical hyperalgesia induced by acid saline Formalin-induced nociception test	Molegro Virtual Docker 6.0.	β-CD improves the anti-hyperalgesic effect of α-TPN; α-TPN-βCD enhances analgesic profile producing a longer-lasting analgesic profile when compared to α-TPN alone; Docking study demonstrated that anti-hyperalgesic effect produced by α-TPN-βCD implies opioid and serotoninergic receptors	Brazil	Oliveira et al., 2016

The table summarizes the main results obtained in research where isolated molecules of essential oils from natural products were used to test for possible antinociceptive activity.

## Results

**Figure 1:**
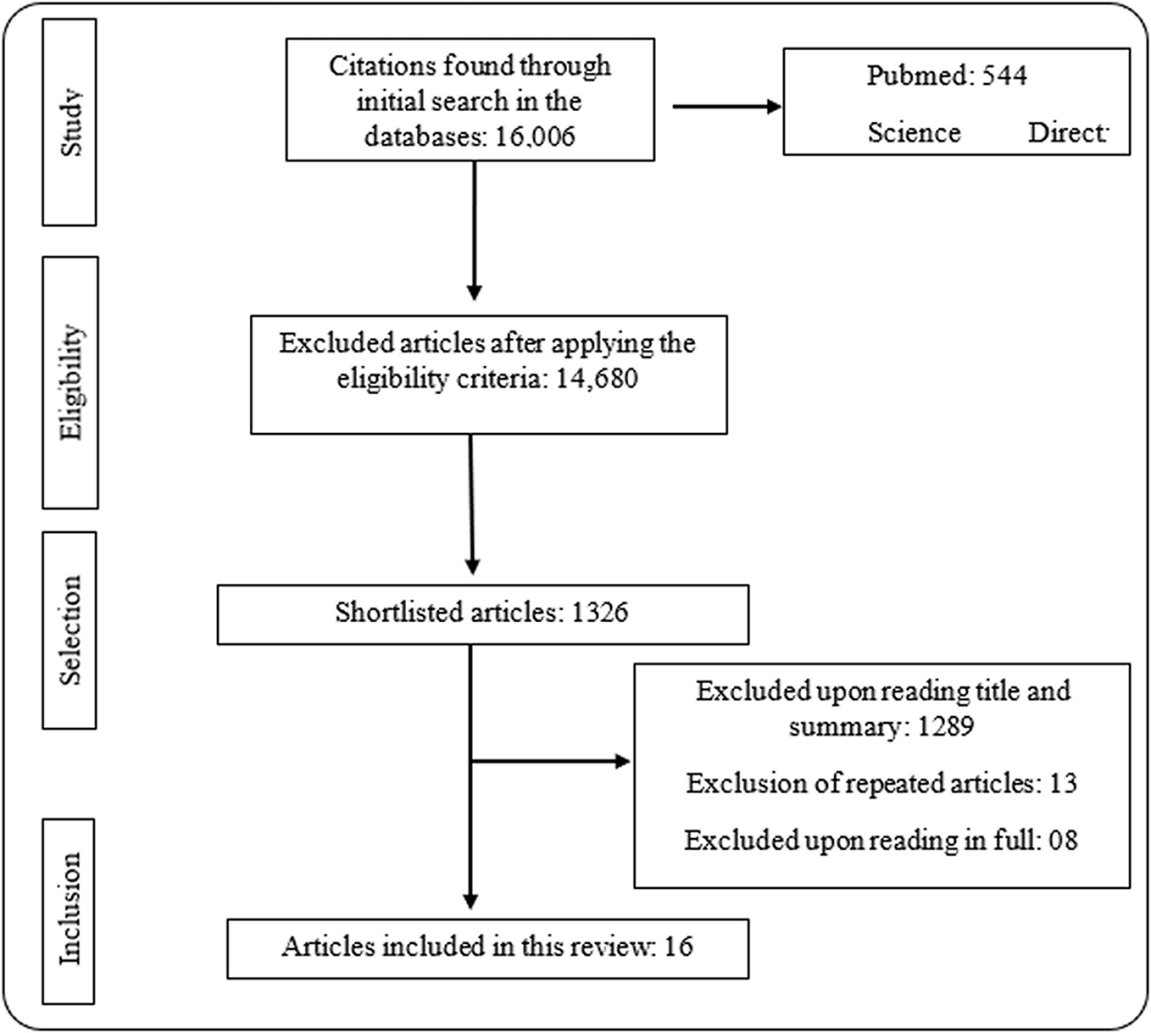
Flowchart of article selection for systematic review. The bibliographic study started with 16,006 articles, which after applying the eligibility criteria, 14,680 remained. Among these, 1326 were selected. A total of 1,289 was excluded after reading the title, 13 were excluded by repetition and 8 were excluded after full reading. In total, 16 articles fit the purpose and were selected for this review.

The initial search of the databases (with the strategies presented in **Table 1**) allowed the identification of 16,006 citations. After filtering the remaining texts included English language articles and various complete free articles; review studies were excluded, leaving 1326 articles, from which a selection based on titles and abstracts for the inclusion criteria mentioned above was performed. At this stage, 1289 articles were excluded, leaving only 37. Upon removal of 13 repeated articles, 24 remained. These studies were subsequently completely read, and finally, 16 articles were selected; 8 articles did not meet all inclusion criteria and were excluded. The selection process can be better visualized in **Figure 1** below, the search flowchart.

The studies identified were concentrated between 2011 and 2019 and are considered current.

There was variability in the study regions for the selected manuscripts, with 75% of the papers coming from the American continents, with 6.25% from North America and 68.75% from South America, 12.5% of the articles originated from the European continent, and 12.5% from the Asian continent.

Of the countries in North America, Mexico represented 6.25% of the publications, in South America, Brazil represented 68.75%, in the European continent, Italy represented 6.25% and Serbia represented 6.25%. In Asia, India and Indonesia stood out, both represented 6.25% of publications.

The most prominent plant families used in the studies identified were *Asteraceae*, (García et al., 2011; Radulović et al., 2015; Leite et al., 2019); *Euphorbiaceae*, (De Oliveira Junior et al., 2017; de Oliveira junior et al., 2018); *Verbenaceae*, (Siqueira-Lima et al., 2017); *Lamiaceae*, (Quintans-Junior et al., 2018), and *Lauraceae* (Sumiwi et al., 2015).

Of the species, *Croton conduplicatus* Kunth prevailed (De Oliveira Junior et al., 2017; de Oliveira junior et al., 2018); with *Vanillosmopsis arborea* Baker (Leite et al., 2019); then *Achillea falcata* L.; (Radulović et al., 2015); *Ageratin glabrata*; (García et al., 2011); *Hyptis pectinata*; (Quintans-Junior et al., 2018); *Cinnamomum; sintoc bl*.; (Sumiwi et al., 2015); and *Grateful Lippia Schauer* (Siqueira-Lima et al., 2017).

Among the phytochemicals studied were α-Terpineol (Oliveira et al., 2016; Gouveia et al., 2018); 3-(5-substituted-1,3,4-oxadiazol-2-yl)-N′-[2-oxo-1,2-dihydro-3H-indol-3-ylidene] propane hydrazide (Kerzare et al., 2016); β-cyclodextrin (CT-βCD) complexed with citronellal (CT) (Santos et al., 2016); (−)-α-bisabolol (Melo et al., 2017; Teixeira, et al., 2017); β-cyclodextrin complexed with farnesol (Silva et al., 2017), and p-Cymene (Santos et al., 2019).

All of the selected studies were non-clinical, the animals used in the studies were Swiss Mice, BALB/c, Wistar, and Sprague Dawley Rats. The tests for evaluation of antinociceptive activity included chemical nociception induction tests: Formalin Test (De Oliveira Junior et al., 2017; de Oliveira Júnior et al., 2018; Leite et al., 2019); Capsaicin (Silva et al., 2017; Leite et al., 2019); Acetic acid (Sumiwi et al., 2015); Glutamate (Silva et al., 2017; Leite et al., 2019); Orofacial formalin (Silva et al., 2017); Saline-induced chronic muscle pain (Siqueira-Lima et al., 2017; Quintans-Junior et al., 2018); Thermal induction tests: Hot Plate Test (Radulović et al., 2015; García et al., 2011; Kerzare et al., 2016; De Oliveira Junior et al., 2017; de Oliveira Júnior et al., 2018); Tail immersion (Radulović et al., 2015); and Mechanical nociceptive induction testing: Von Frey (Santos et al., 2016; Gouveia et al., 2018 Santos et al., 2019).

The *in silico* programs used for docking studies were; Molegro Virtual Docker (Oliveira et al., 2016; De Oliveira Junior et al., 2017; Siqueira-Lima et al., 2017; de Oliveira Júnior et al., 2018; Gouveia et al., 2018; Leite et al., 2019; Santos et al., 2019); Sybyl^®^X software suite (García et al., 2011), Vlife MDS (Kerzare et al., 2016); AutoDock Vina (Santos et al., 2016); Hex Protein Docking (HEX) (Teixeira et al., 2017; Melo et al., 2017); and AutoDock 4.2 software in PyRx 0.9 (Silva et al., 2017).

The molecular targets/complexes used in the docking studies were Nitric Oxide Synthase (Gouveia et al., 2018), COX-2 (García et al., 2011; Sumiwi et al., 2015; Kerzare et al., 2016); GluR2-S1S2 (Santos et al., 2016); TRPV1 (Teixeira et al., 2017; Melo et al., 2017); β-CD complex (Silva et al., 2017); CaV1, CaV2.1, CaV2.2, and CaV2.3 (Santos et al., 2019); 5-HT receptor, Delta receptor, Kappa receptor, and MU (µ) receptor (Oliveira et al., 2016); Alpha Adrenergic, Opioid, and Serotonergic receptors (Siqueira-Lima et al., 2017); Muscarinic receptors and GABA_A_ (De Oliveira Junior et al., 2017; de Oliveira Júnior et al., 2018); Opioid and Serotonin receptors (Quintans-Junior et al., 2018); 5-HT 3 and M2 receptors (Leite et al., 2019).

The study results were analyzed separately and can be seen in **Table 2**, along with other information from the articles.

## Discussion

With technological development and advances in understanding the pathophysiological bases of pain/nociception, alternative screening methods for naturally occurring compounds have gained specified target approaches. Various models have been developed and tested against natural compounds. Previous studies, including a systematic review, showed the potential antinociceptive effect of essential oils and their phytoconstituents, especially monoterpenes. However, most of the reports of these investigations present obtained by experimental animal assays (Guimarães et al., 2012; Sá et al., 2017). Through this review, although scarce, studies coupling molecular anchoring with EO antinociceptive activity and their constituents (monoterpene - sesquiterpene) (**Table 4**) allow observations concerning the obtained molecular hits. This increases the chances of finding candidate molecules for therapeutic use. Certain species were studied for possible antinociceptive activity with the aid of molecular coupling. For these, it was possible to predict the preferential orientation (when linked together to form a stable complex) of one molecule to another, and further elucidated molecular interactions.

### *Vanillosmopsis arborea* Baker

*Vanillosmopsis arborea* Baker (Asteraceae) is a native plant to northeastern Brazil, especially the state of Ceará (Matos et al., 1988). There are insufficient studies reporting the biological effects of the essential oil extracted from this plant, but its leishmanicidal (Colares et al., 2013), gastroprotective (De Oliveira et al., 2009), and antimalarial (Mota et al., 2012) activities have been described. According to De Oliveira et al. (2009), an analysis by gas chromatography coupled to mass spectrometry (GC/MS) of *V. arborea* stem bark essential oil (EOVA) evidenced the existence of α-bisabolol (70%), and also α-cadinol (8.4%), elemicin (6.21%), β-bisabolene (4.46%), δ-guaiene (2.31%), β-cubebene (1.76%), and estragole (1.08%).

To increase the bioavailability and pharmacological properties of essential oils, complexation with β-cyclodextrin is very useful (Santos et al., 2016). Cyclodextrins are cyclic oligosaccharides presenting a hydrophobic center that complexes with molecules yet improves water solubility and reduces toxic effects (Oliveira et al., 2009; Abril-Sánchez et al., 2019). EOVA and its form as complexed with β-cyclodextrin (EOVA-pCD) at a dose of 50 mg/kg, i.p. reduced orofacial nociception induced by various stimuli, as well as in a model of temporomandibular joint dysfunction caused by the administration of formalin. The study also suggested that EOVA modulates type 1 vanilloid transient potential receptors, yet without interacting with the glutamatergic inhibitory pathway. Fos protein expression in the dorsal horn of the spinal cord was decreased by pCDEVA, inferring a reduction in pain-sensitive neuronal activation. In the same study, (through molecular docking) the researchers observed favorable interactions between bisabolol, the major constituent of EOVA, the 5-HT3 receptor, and type 3 muscarinic receptors (Leite et al., 2019).

### Citronellal

Citronellal (**Table 5**, ID 01) is a monoterpene isolated from aromatic plants of the genus Cymbopogon (Quintans-Junior et al., 2008). Several pharmacological activities have been described for this compound, which acts as an anti-atherosclerotic (Lu et al., 2019), antifungal (Wu et al., 2016), anti-inflammatory (De Santana et al., 2013), and anticonvulsant (Melo et al., 2011).

**Table 4:** Interactions observed in docking studies involving antinociceptive activity.

Ligand	Molecular target	Interacting amino acids	Reference
Citronellal	GluR2-S1S2	Arg96, Ser142 e Thr143.	Santos et al., 2016
α-Terpineol	Nitric Oxide Synthase	Thr324, Trp325 e Ile327.	Gouveia et al., 2018.
(−)-α-bisabolol	TRPV1	Ala680, Gly683, Asn687.	Teixeira et al., 2017.
(−)-α-bisabolol	TRPV1	Ile695, Ser972, Leu973 and Lys969.	Melo et al., 2017
p-Cymene	CaV1, CaV2.1, CaV2.2 and CaV2.3	Glu84, Glu87, Ala88, Val91, Met144.	Santos et al., 2019
α-Terpineol	5-HT receptor Delta receptor Kappa receptor MU receptor	Asp129 and Cys133. Asp128. Leu67 and Val63. Asp147.	Oliveira et al., 2016
(−)-α-bisabolol	5-HT3 and M2 receptors.	Tyr64, Arg65, Thr154, Trp156 and Glu209. Asp103, Tyr104, Ala194 and Tyr403.	Leite et al., 2019.
Camphor, transcarophyllene and bicyclogermacrene	Alpha adrenergic, µ Opioid, and 5-HT.	Arg14, Tyr15, Ile18 and Thr19. Asp135, Val136, Thr140, Phe340 and Phe341. Gln124, Tyr148, Val236, His297, Trp318.	Siqueira-Lima et al., 2017.
Eugenol	COX-2	Val116, Arg120, Val349, Leu352, Tyr355, Phe518, Met522.	Sumiwi et al., 2015.
b-FNA, Germacrene D, Caryophyllene oxide, Linalool and β-caryophyllene	Opioid and serotonin receptors.	Thr134 and Val201.	Quintans-Junior et al., 2018.
3-(5-substituted-1,3,4-oxadiazol-2-yl)-N′-[2-oxo-1,2-dihydro-3H-indol-3-ylidene]propane hydrazides derivatives	COX-2	Pro127, Tyr373, Gly536, Gln374, Arg376 and Ser541.	Kerzare et al., 2016.
1,8-Cineole, Caryophyllene oxide, p-Cymene, Spathulenol,	Muscarinic receptors and GABAA.	Not described.	de Oliveira Júnior et al., 2018.
10-benzoiloxi-6,8,9-isobutirato de tri-hidroxi-timol	COX-2	Not described.	García et al., 2011.
trans-sabinol and trans-sabinyl acetate	AChE	Ser200, Glu327, His440, Phe330 and Trp 84.	Radulović et al, 2015.
B-cyclodextrin complexed with Farnesol	β-CD complex	Not described.	Silva et al., 2017.

The table summarizes the ligands and molecular targets used in molecular docking research, as well as the residues that showed interaction with the compounds.

**Table 5:** Main docking compounds used in the articles included in this review.

ID	Name	Structure	Reference	ID	Name	Structure	Reference
01	Citronellal	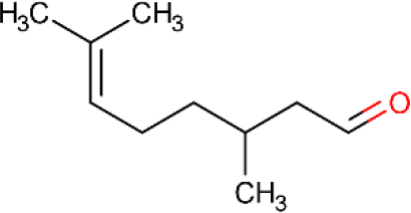	Santos et al., 2016	09	Linalool	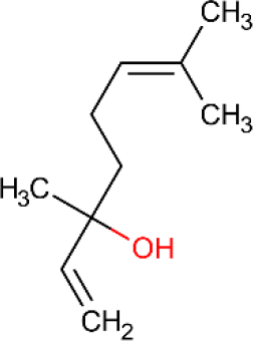	Quintans-Junior et al., 2018.
02	α-Terpineol	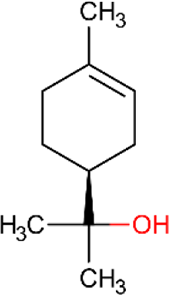	Gouveia et al., 2018. Oliveira et al., 2016	10	β-caryophyllene	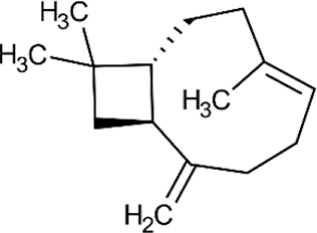	Quintans-Junior et al., 2018.
03	(−)-α-bisabolol	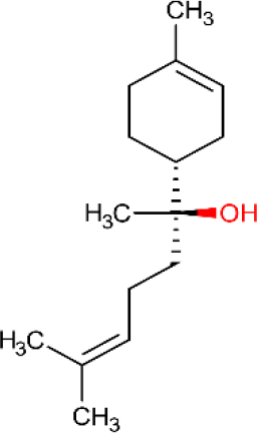	Teixeira et al., 2017 Melo et al., 2017. Leite et al., 2019.	11	1,8-Cineole	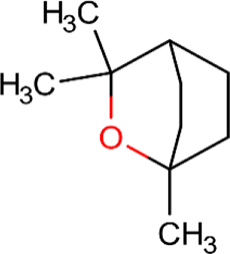	de Oliveira Júnior et al., 2018.
04	p-Cymene	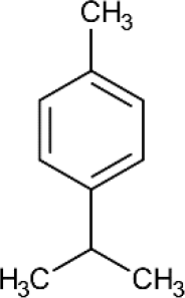	de Oliveira Júnior et al., 2018.	12	Spathulenol	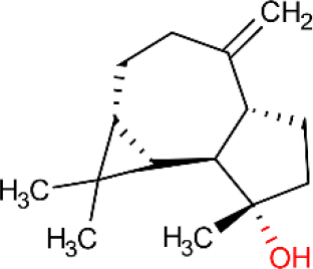	de Oliveira Júnior et al., 2018.
05	Camphor	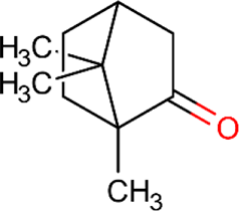	Siqueira-Lima et al., 2017.	13	trans-sabinol	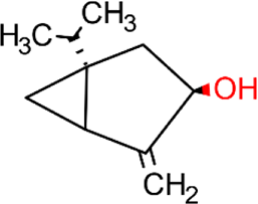	Radulović et al, 2015.
06	Eugenol	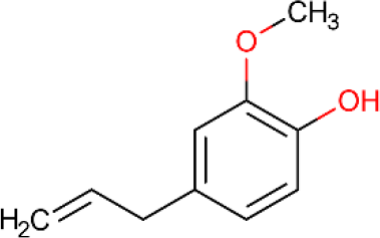	Sumiwi et al., 2015.	14	sabinyl acetate	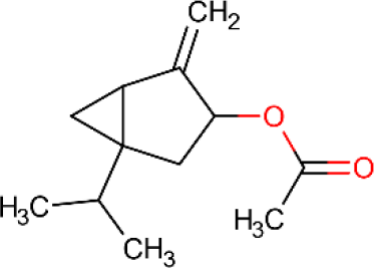	Radulović et al, 2015.
07	Germacrene D	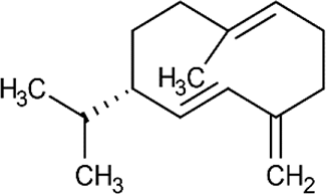	Quintans-Junior et al., 2018.	15	Farnesol	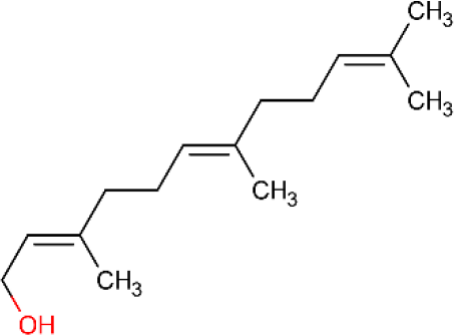	Silva et al., 2017.
08	Caryophyllene oxide	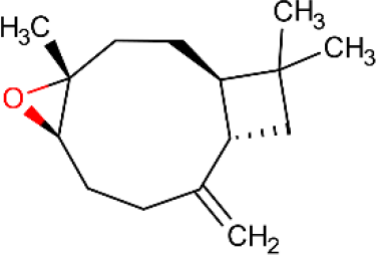	Quintans-Junior et al., 2018.				

This table summarizes the structure of compounds that interact with protein targets in docking studies.

Despite the known effects of isolated monoterpenes, the complexation of monoterpenes with β-cyclodextrin has produced more promising results for treating pain and inflammation (Quintans-Junior et al., 2013; Nascimento et al., 2014; Silva et al., 2016). In a chronic non-inflammatory muscle pain model, chronic treatment using a citronellal/β-cyclodextrin complex at a dose of 50 mg/kg, p.o. presented longer-lasting antihyperalgesic effects than citronellal alone. The results can be explained by an increase in c-Fos protein expression in the ventrolateral periaqueductal gray and rostroventral medullary areas of the brain and reductions in this activity in the superficial dorsal horn, suggesting inhibitory modulation of descending pain pathways (Santos et al., 2018). In a molecular docking study, a favorable energy bond between citronellal and the structure GluR2-S1S2J, an ionotropic glutamate receptor responsible for painful stimulus propagation was observed (Santos et al., 2016).

### α-Terpineol

**α-**Terpineol (**Table 5**, ID 02) is a monoterpene present in the essential oils of *Ravensara aromatica, Melaleuca qinquenervia*, and mainly, species of the genus Eucalyptus (Elaissi et al., 2010). Studies show the clinical potential of this compound to treat various types of pain (Quintans-Júnior et al., 2011; De Oliveira et al., 2012; Oliveira et al., 2016; Safaripour et al., 2018).

In an experimental model of cancer pain consisting of subcutaneous implantation (in the plantar region) of tumor cells in mice, subcutaneous treatment with α-terpineol (12.5, 25, and 50 mg/kg, sc) promotes an anti-hyperalgesic effect and seems to reduce allodynia in these painful conditions, without promoting myorelaxant effects. The effect may be related to antioxidant capacity and reduced inducible nitric oxide synthase (iNOS) levels in the tumor microenvironment. Molecular docking corroborates these results, since it was observed that the monoterpene binds to iNOS in the same regions as N-Nitroarginine methyl ester (an iNOS inhibitor) (Gouveia et al., 2018).

A complex containing α-terpineol and cyclodextrin (αTPN-pCD) at doses of 25, 50, and 100 mg/kg, p.o. reduced mechanical hyperalgesia caused in an animal model of fibromyalgia. In the same study, the involvement of the opioid and serotonergic system in the analgesic activity was observed both through the use of pharmacological antagonists in animals, and when employing molecular docking (Oliveira et al., 2016).

### α-Bisabolol

α-Bisabolol (**Table 5**, ID 03) is a monocyclic sesquiterpene extracted from the essential oils of various plants, mainly the flowers of *Matricaria chamomilla* and the bark of *Vanillosmopsis arborea* Baker (Kamatou and Viljoen, 2010; Inocencio Leite et al., 2014). Several biological effects have been described for α-bisabolol including neuroprotective (Fernandes et al., 2019), antiparasitic (de Menezes et al., 2019), anticancer (Wu et al., 2018), and antibacterial activity (de Sousa Oliveira et al., 2017).

In an orofacial antinociceptive evaluation of α-bisabolol, the non-clinical efficacy of this compound when administered systemically or topically was confirmed. In a temporomandibular joint dysfunction model, α-bisabolol reduced nociceptive orofacial rubbing behavior in rats. The pharmacological effects occur independently of ATP-sensitive nitrergic systems, opioids, and potassium channels. Molecular docking revealed a strong interaction and the absence of significant electrostatic repulsion between α-bisabolol and the TRPA1 receptor, suggesting possible antagonism (Melo et al., 2017). Texeira et al. (2017), observing TRPV1 antagonism in molecular docking studies, revealed that nanocapsules containing α-bisabolol present antinociceptive activity in an orofacial pain model caused by administration of hypertonic saline into the mouse cornea.

Still talking about terpenes and TRPV1 receptors, Jansen et al. (2019), found that myrcene has a significant participation in calcium inflows mediated by the TRPV1 receptor. Myrcene has been identified as an analgesic in previous studies, demonstrating its anti-nociceptive potential in mice (Rao et al., 1990). Responses to myrcene showed total dependence on the presence of TRPV1 and were effectively blocked by capsaicin, a TRPV1 antagonist. Patch Clamp studies showed that myrcene is indeed an effective TRPV1 ligand and in cells that contain high TRPV1 receptor densities, leading to intracellular calcium release. While for many pain applications there is a focus on TRPV1 antagonists, other applications depend on the chronic agonism of TRPV1, leading to the desensitization of TRPV1 at the cellular level and the induction of neuronal cell death by large inflows of calcium and sodium through the channel (Derry et al., 2017). Preliminary molecular coupling studies suggest that myrcene is interacting hydrophobically, non-covalently, and in a way that does not depend heavily on reactive cysteines. Tyr 554 is involved in the binding site, which has also been implicated in capsaicin binding (Elokely et al., 2016) and forms part of the S4-S5 loop between the fourth and fifth transmembrane domains of TRPV1 (Boukalova et al., 2010).

Like myrcene, camphor also desensitizes TRPV1 receptors, but in a more rapidly and completely way than capsaicin, it activates TRPV3 and inhibits TRPA1, correlating to its analgesic properties (Xu et al., 2005).

The endocannabinoid system involves the central and peripheral nervous systems. It is involved in inflammatory and pain processes (Rodriguez de Fonseca et al., 2005). The endocannabinoid system appears to work both independently and synergistically (Mallat et al., 2011; Baron, 2018). Cannabidiol (CBD) is the second major cannabinoid and has much lower affinity for CB1 and CB2 receptors as compared to Δ9-tetrahydrocannabinol (THC), and it acts as a non-competitive CB1 and CB2 receptor antagonist (Thomas et al., 2007). CBD has additional actions that may account for its anti-inflammatory and analgesic effects including TRPA1 agonist, TRPV1 agonist (similar to capsaicin, although without the noxious side effects), TRPM8 antagonist (De Petrocellis et al., 2011; Baron, 2018).

Jansen et al. (2019) show that myrcene and CBD share elements of a binding site and can influence one another physiologically. Their data suggest that several minor cannabinoids discriminate between TRPV1, TRPA1, and TRPM8 (Starkus et al., 2019). In CBD docking studies, a binding pocket partially over-lapping with that of Myrcene was identified and the best scoring pose showed a docking score of −26.5 kcal/mol. In this site, Thyr 554 and Arg 491 are important, as for Myrcene. The remaining residues implicated in CBD binding show both similarities and differences to the Myrcene site (Jansen et al., 2019).

### Croton conduplicatus Kunth

*Croton conduplicatus* Kunth is a Brazilian medicinal plant, belonging to the Euphorbiaceae family, called as “quebra-faca” by the locals; it is found in South America. In the Brazilian northeast, folk medicine uses its leaves to treat stomach pain (Cartaxo et al., 2010). GC/MS analysis of *Croton conduplicatus* Kunth stem bark (essential oil) revealed a majority presence of the terpenoids (E)-caryophyllene (13.72%), caryophyllene oxide (**Table 5**, ID 08) (13.15%) and camphor (8.25%). The essential oil presents central and peripheral antinociceptive activity *via* possible involvement of K_ATP_ channels and muscarinic receptors, yet without participation of opioid receptors. In the same study, a satisfactory link was observed in molecular docking between the constituent terpenoids and muscarinic receptors (M2, M3, and M4) (De Oliveira Júnior et al., 2017).

Chemical analysis of *Croton conduplicatus* Kunth leaf essential oil revealed 1,8-cineole (**Table 5**, ID 11) (21.42%), p-cymene (12.41%), spathulenol (**Table 5**, ID 12) (15.47%), and caryophyllene oxide (12.15%) as the main constituents. The oil presents antinociceptive activity equal to that of the leaves, yet involvement of the opioid system is observed. In molecular docking, the interactivity of spathulenol and caryophyllene oxide with opioid and muscarinic receptors was verified (de Oliveira Júnior et al., 2018).

### Complexed β-cyclodextrin (βCD)—*Lippia grata essential oil*

*Lippia grata* Schauer is a shrub widely distributed in northeastern Brazil. Folk medicine uses it to treat pain conditions (Viana et al., 1981). Terpene-rich *L. grata* (LG) leaf essential oil is known to promote an orofacial analgesic profile. Since it is known that the therapeutic analgesic effects of certain EO and its constituents can be enhanced by forming a β-cyclodextrin (βCD) inclusion complex (Oliveira et al., 2016), Siqueira-Lima et al., 2017 evaluated the antinociceptive effect of a β-cyclodextrin (βCD) complex with *Lippia grata* essential oil in an animal model of chronic musculoskeletal pain. The model mimicked painful muscle diseases such as fibromyalgia (FM), which is a rheumatic disease characterized by widespread chronic pain and is difficult to treat. The low clinical efficacy and side effects of current treatments make therapeutic adherence more difficult, and the lack of knowledge about the pathophysiology of FM complicates the development of new drugs. Animal models are an alternative for reproducing FM symptoms. In a model of chronic non inflammatory muscle pain, caused by saline acid injection in the mouse gastrocnemius, oral administration of LG-βCD produced excellent antihyperalgesic activity. LG-βCD also did not alter muscle strength, discarding the chance of reduced motor performance, common in some CNS drugs. An assay to evaluate possible antagonist involvement of opioid, serotoninergic, and noradrenergic pathways revealed that the antihyperalgesic effect of LG-βCD may involve serotonergic and opioid receptors, indicating probable participation of the inhibitory modulation system of descending pain. This was partially supported by the *in silico* study. The receptor structures were obtained from Protein Data Bank (PDB). The investigated receptors, by comparing binding energy with ligands; camphor (**Table 5**, ID 05), trans-caryophyllene, bicyclogermacrene (**Table 5**, ID 07) (major LG compounds) were alpha-adrenergic, μOpioid, and 5HT. *In silico* target validation analysis favored the understanding of the drug-receptor interaction, and confirmed the observed result within *in vivo* tests, demonstrating that camphor and E-caryophyllene bind to the alpha-adrenergic receptor, while bicyclogermacrene binds with moderate energy to 5-HT and μOpioid receptors (Siqueira-Lima et al., 2017).

### *Hyptis pectinata* Leaf Essential Oil

*Hyptis pectinata* belongs to the Lamiaceae family, which is characterized by the presence of strongly aromatic plants, present in North and South America, mainly in tropical areas. In traditional medicine, *H. pectinata* extracts have been used as medicinal teas in pain treatment. The anti-inflammatory and analgesic profile of *H. pectinata* has been reported in many studies in the literature. The analgesic effects of *H. pectinata* EO occur due to the terpenoid compounds, such as β-caryophyllene (**Table 5**, ID 10), caryophyllene oxide, linalool (**Table 5**, ID 09), and limonene (McNeil et al., 2011). The antinociceptive effect of β-caryophyllene occurs due to its cannabimimetic effects (Gertsch et al., 2008). The complex containing β-caryophyllene and β-cyclodextrin has anti-hyperalgesic properties in the model of chronic muscle pain *via* inhibition of c-Fos expression in the lumbar spinal cord (Quintans-Júnior et al., 2016).

The EO of *H. pectinata* complexed with β-cyclodextrin is able to increase the analgesic effect, as well as extend its duration (Menezes et al., 2015). Quintans-Junior et al. (2018) evaluated the capacity of a nanostructured thermoreversible subcutaneous hydrogel aggregated with *H. pectinata* essential oil (NE-EOH) to promote long-term antihyperalgesic effect in a fibromyalgia (FM) animal model. This formulation (containing *H. pectinata* essential oil) produced a lasting antihyperalgesic effect in a non-inflammatory muscle pain model in mice. NE-EOH produces analgesia through CNS inhibitory mechanisms. Through a molecular anchoring study, it was possible to predict that this antihyperalgesic response involves central pain inhibitory pathways and endogenous serotonin and opioid neurotransmitters.

To evaluate the binding ability of NE-EOH to the tested targets (serotonin and opioid receptors), coupling analysis using MolDock was performed, taking into account the binding energy of the major *H. pectinata* EO compounds (β-caryophyllene, caryophyllene oxide, germacrene D, and linalool) with μ-OR β-FNA receptors and 5-HT1B dihydroergotamine. A hydrogen bond was observed between the epoxy portion of caryophyllene oxide and 5-HT1B (Thr 134), an equal interaction could be seen for dihydroergotamine. Caryophyllene oxide and germacrene D presented the lowest binding energies of the studied secondary metabolites, suggesting that germacrene D and caryophyllene oxide possibly contributes in the antihyperalgesic activity of NE-EOH, *via* the μ-OR pathway (Quintans-Junior et al., 2018).

### *Cinnamomum sintoc bl* Bark Essential Oil (sintoc)

Sintoc (*C. sintoc bl*) is a plant grown in Indonesia, Malaysia, and Thailand and used in folk medicine to treat swelling (inflammation). Sumiwi et al., 2015 investigated the anti-inflammatory and analgesic activity of *Cinnamomum sintoc bl* (sintoc) bark essential oil, (inhibiting the enzyme COX-2), using animal models, together with molecular coupling to predict the interaction of sintoc compounds with COX-2. Eugenol (a constituent of sintoc (**Table 5**, ID 06)) presented a good visual interaction with COX-2. The phenol part of eugenol forms hydrogen bonds with Gly 526 and Met522 of COX-2, as well as the naproxen phenol. However, eugenol presents no electrostatic interaction between carboxyl and ammonium ions, such as naproxen carboxyl groups with Arg120 ammonium ions.

In the *in vivo* tests, the essential oil of sintoc bark presented analgesic activity in the acetic acid-induced abdominal writhing test and anti-inflammatory activity in the carrageenan-induced paw edema test. In a previous molecular anchorage study, the results revealed that isoeugenol can effectively inhibit the enzymatic activity of cyclooxygenase and lipoxygenase. Isoeugenol anchored at the active site with an orientation similar to that of indomethacin (Zarlaha et al., 2014).

### Ageratina glabrata

Species of the genus Ageratina belong to the Asteraceae family. In general, this genus is known to have therapeutic activities, among which its analgesic effects stand out (Mandal et al, 2005; Chakravarty et al., 2011).

*Ageratina glabrata* (Kunth) is commonly known in Mexico as “chamizo blanco” or “hierba del coup”. Folk medicine reveals the use of this plant for pain relief. The literature describes the presence of flavones, thymol derivatives, and other phenolic terpenoids in its chemical composition (Vivar et al., 1971; Bohlmann et al., 1977; Guerrero et al., 1978).

Using the hot plate test, García et al. (2011), verified the presence of analgesic effect in a group of animals treated at 100mg/kg with *A. glabrata* leaf extract. The results, because of the duration and pain suppression characteristics, and is similar to those observed in the positive control treated with meloxicam, suggest that the molecular mechanism involved may be *via* cyclooxygenase (COX).

After isolation and purification, Garcića et al. (2011) identified the presence of trihydroxy thymol 10-benzoyloxy-6,8,9-isobutyrate, which became the molecule from *A. glabrata* extract chosen to conduct a docking study using the Sybyl^®^ software. The protein was downloaded from the Protein Data Bank with code 3LN0. Both meloxicam and the derivative was successfully docked in the same position and orientation at the PDB ligand, at the active site of the COX-2 enzyme. The binding and formation of the ligand-enzyme complex were in agreement with the crystallographic structure, showing the potential of this derivative to interact with COX-2 and promote the analgesic effect evidenced in the *in vivo* thermal nociception model.

### *Achillea falcata* L.

*Achillea falcata* L. is an endemic Mediterranean species widely considered for its pharmacological effects, and traditionally used to treat fever, stomach pain, and hemorrhoids (Aburjai et al., 2007; Alzweiri et al., 2011).

Radulović et al. (2015) have been able to provide clear evidence that *A. falcata* is capable to produce trans-sabinol (**Table 5**, ID 13) and some of its esters. Its constituents are biosynthesized and accumulate in the aerial parts and roots of the plant (Kürkçüoğlu et al., 2003; AburjaiHudaib, 2006). The compounds formate and tiglate have been recently discovered (Radulović et al., 2015).

*Achillea falcata* essential oil characterization (aerial parts and roots) was performed near the city of Ma’loula, Syria, revealing the presence of two principal constituents, trans-sabinol (19.1%) and trans-sabinyl acetate (**Table 5**, ID 14) (11.4%), as well as their rare esters. Using different models such as abdominal contortions and hot plate, Radulović et al. (2015) screened to investigate the analgesic potential of trans-sabinol and its esters.

Due to the structural similarity of these compounds with rivastigmine, an acetylcholinesterase (AChE) inhibitor, there was an interest in verifying *in silico* (molecular docking), the ability of these esters to interact with AChE. The active site of AChE is known to be deep within the enzyme. The residues of Ser200, His440, and Glu327, located at the bottom depth, form the catalytic triad and participates directly in hydrolysis of acetylcholine (Sussman et al., 1991; Millard et al., 1999).

All of the esters found energetically favorable coupling poses, placing the ligands at the catalytic site, and suggesting that the compounds may indeed approach the amino acid residue triad. The most favorable anchorage position was achieved by trans-sabinyl. This compound was placed with the electrophilic ester/bond group near the residues of Ser200, Glu327, His440, Phe330, and Trp 84, which are relevant for AChE function (Sussman et al., 1991; Millard et al., 1999). The ligand disposal indicates that it might interact with the enzyme allowing covalent modification. The other esters came with a similar outcome, and the calculated binding energies suggests that trans-sabinyl tiglate and senecioate initially bind more strongly to the enzyme (8.5 kcal/mol), while trans-sabinyl formate likely has the lowest affinity for AChE (Radulović et al., 2015).

The mentioned compounds also presented antinociceptive activity in two different animal models. Trans-sabinol promoted a reduction in the animal’s response to thermal stimuli in the hot plate test, and a reduction in the abdominal writhing test response as well. When subjected to the hot plate test at the dose of 50 mg/kg, trans-sabinol was able to reach its maximum effect after 15 min. In the same test, time and dose, trans-sabinyl tiglate increased the residence time by 140% (Radulović et al., 2015). These results, associated with molecular anchoring tests, indicate that trans-sabinol and its esters interact with different targets and influence both the periphery and the central nervous system, been capable to promote a considerable antinociceptive effect.

### β-Cyclodextrin Complexed With Farnesol

Farnesol (FAR) (**Table 5**, ID 15) is a naturally occurring sesquiterpene alcohol known to exhibit multiple functions including Ca^2+^ channel inhibition (Endo et al., 2011; Khan and Sultana, 2011; Santhanasabapathy et al., 2015), an important target for drugs used in chronic pain (Clark et al., 2016). This sesquiterpene has been shown to possess anti-inflammatory and analgesic properties but without considerable neurotoxicity in the brain of adult mice (De Oliveira Junior et al., 2013).

A molecular docking study was performed to predict the likely interaction between β-cyclodextrin (β-CD) and FAR. It was observed that of the ten conformations generated by FAR with β-CD there were stable fittings (forming complexes) with the lowest energy value being - 3.45 kcal/mol. The presence of hydrogen bonds between farnesol and β-CD was also observed.

In the formalin test, administration of FAR (50 mg/kg) was able to reduce face rubbing time (p < 0.05); FAR at 100 mg/kg, and FAR + β-CD in 50 and 100 mg/kg doses reduced this behavior in both phases of the formalin test (p < 0.001). From the results, FAR + β-CD was significantly more effective in reducing pain behavior than FAR alone (Silva et al., 2017).

Experiments using a capsaicin-induced orofacial pain model showed that administration of FAR (50 mg/kg) also reduced face rubbing time (p < 0.05), yet FAR at 100 mg/kg, and the FAR + β-CD complex at doses of 50 and 100 mg/kg reduced this behavior more effectively (p < 0.001). Two targets act in this pain pathway, TRP receptors, and Ca^2+^ channels, thus suggesting their inhibition by the compounds (Silva et al., 2017).

Glutamate tests revealed that FAR, and FAR + β-CD at doses of 50 and 100 mg/kg significantly inhibited nociception (p < 0.0001). Statistical differences between FAR + β-CD at 100 mg/kg dose, and doses of isolated FAR at 50 and 100 mg/kg (p < 0.0001) indicated once again that the complex is more effective (Silva et al., 2017).

In a study on the mechanism of action, both isolated FAR and FAR + ondansetron presented statistical differences (p < 0.0001), demonstrating potential interactions with serotonergic receptors. The 5-HT3 receptor plays a pro-nociceptive role, mediating descending excitatory pathways in the spinal cord dorsal horn (Azimaraghi et al., 2014).

Overall, FAR + β-CD demonstrated a better pharmacological effect than the active compound alone. FAR + β-CD reduced orofacial pain behavior, which according to the investigation of the mechanism of action, was potentially mediated by interaction with 5-HT3 receptors. The inclusion complex that contains FAR + β-CD, therefore, suggest having therapeutic potential in the treatment of some types of dysfunctional pain, such as orofacial pain.

### 2-Oxoindolin-3-ylidene-3-(5-substituted phenyl-1,3,4-oxadiazol-2-yl) Propanehydrazide Derivatives

The hybrid approach involves the development more effective synergistic molecules by hybrid mixing of two or more already active biomolecules to produce new derivatives that have better pharmacological activity (Gediya and Njar, 2009).

The target portions selected for the formation of 2-oxoindolin-3-ylidene-3-(5-substituted phenyl-1,3,4-oxadiazol-2-yl)-propanehydrazide hybrids were based on studies revealing the analgesic and anti-inflammatory properties of indole and oxadiazole nuclei (Pandeya et al., 1998; Wagle et al., 2008; Chikhale et al., 2009; Jayashankar et al., 2009; Singh et al., 2010; Chaluvaraju et al., 2011).

Fifteen different hybrids of indole and oxadiazole were synthesized. *In silico* determinations of potentials compound–receptor interactions were performed through molecular coupling studies of the ligands at the cyclooxygenase (COX) site (Chikhale et al., 2015). Docking of these derivatives with COX-2 (PDB code 4Z0L) was performed using Vlife MDS Molecular Modeling 4.3.1 software. One of the reasons for choosing this enzyme was that its crystallographic structure already provides complexing with an indole derivative, and it can act as a reference molecule for coupling. As well, the animal model used also favored the choice. Anchoring was performed for all of the synthesized compounds. Three compounds, 50 (p-OH), 51 (p-CH3), and 53 (o-OCH3, m′-OCH3) exhibited the best activity, with 50 and 51 the most active. As energias de 50 (−4,44) e 51 (−4,37) foram as mais altas da série de derivados sintetizados, comparáveis à indometacina, o medicamento padrão, com uma pontuação de 4,47. Compound 50 was shown to bind at the active site of COX-2, forming hydrogen bonds at GLY536 and TYR373. Hydrophobic interactions were found mainly at GLN374 and ARG376. Compound 51 also stood out for interacting at the COX-2 active site, forming two hydrogen bonds at ASN375 (Kerzare et al., 2016). The presence of methyl or hydroxyl groups, as well as a 1,3,4-oxadiazole nitro substitution, increased the activity. The nesting of these engineered molecules demonstrated their entry into a deep region of the enzyme. Thus, the presence of an indole ring and oxadiazole in the molecule are considered beneficial for activity, but the presence of halogens such as chlorine or fluorine reduces activity.

Was evaluated the analgesic activity of the synthesized compounds using the hot plate test. They were tested at an oral dose of 100 mg·kg^−1^, and compared to indomethacin at a dose of 100 mg·kg^−1^ (v.o.), the tested compound series showed analgesic activity after 90 min ranging from 25.13% to 84.11%. The results revealed compounds 50, 51, and 52 (*m-*NO_2_) as presenting good analgesic activity, while compounds 49 and 61 (o-F) presented intermediate activity, and compounds 56 (p-COCH3) and 57 presented lesser activity as compared to the medicinal standard. The results indicate that compounds possessing electron-withdrawing groups with *para* and *meta* substituents can increase analgesic activity, while electron donor groups decrease activity (Kerzare et al., 2016).

### p-Cymene

P-cymene (**Table 5**, ID 04) is a monoterpene found in the essential oil of approximately 100 herb species, and also present in over 200 types of food (Selvaraj et al., 2002). Its many biological effects, such as anti-inflammatory and analgesic activity, have been studied and demonstrated in various parts of the world (Bonjardim et al., 2012; De Souza Siqueira Quintans et al., 2013; Quintans-Junior et al., 2013; De Santana et al., 2015).

As a potential alternative to cancer-associated pain, Santos et al. (2019) evaluated the effects of p-cymene on animal models of Sarcoma 180 (S180) induced nociception and investigated how it may act to promote such effect.

The animals submitted to the sarcoma-causing agent received the treatment with p-cymene at doses of 12.5, 25 or 50 mg/kg subcutaneously for 15 days in a row. The rats were evaluated for sensitivity to mechanical stimulation using the von Frey test on the tumor-bearing paw. Four measurements were taken between 3-min intervals to verify the stimulus intensity. It was seen that p-cymene at a dose of 50 mg/kg was able to reverse the hyperalgesic graph, starting from the 11th to the 15th day, with 60.4% of inhibition, equivalent to morphine, which similarly caused a decrease in animal perception (Santos et al., 2019).

The molecular interactions between p-cymene and the various voltage-dependent calcium channel subtypes were analyzed by docking studies, using p-cymene, nicardipine, ω-conotoxin GVIA, ω-agatoxin IVA, and N-triazole oxindole as ligands. The Protein Data Bank provided the macromolecules for this study, which were CaV1 calcium channel (type L) (PDB ID 5GJV), CaV2.1 calcium channel (type P/Q) (PDB ID 3BXK), CaV2.2 calcium channel (type N) (PDB ID 3DVE) and CaV2.3 calcium channel (type R) (PDB ID 3BXL). All ligands were subjected to molecular anchoring using the MolDock algorithm (Thomsen and Christensen, 2006; Santos et al., 2019).

When p-cymene interacted with CaV1, CaV2.1, CaV2.2, and CaV2.3 calcium channels, the respective negative energy values of −60.118, −59.60, −49.55, and −59.95 kcal/mol suggested that binding between the targets is both favorable and likely occurs since such negative values suggest lower energy expenditure to assume a more stable interaction (Du et al., 2016). These voltage-dependent calcium channels can be found at presynaptic terminals and participate in the release of neurotransmitters, such as substance P, glutamate, and CGRP (Lee, 2013). The density of the calcium stream was significantly reduced by p-cymene. It is known that direct inhibition of calcium channels alone by exogenous ligands may cause antinociception (Freitas et al., 2018). In addition, this mechanism of action is equivalent to certain existing drugs that are currently used to treat chronic pain, such as gabapentin (Santos et al., 2019; Catterall and Swanson, 2015).

We observed that many studies covered in this review used molecular coupling to investigate the mechanism of action of several compounds, one of the objectives of molecular docking studies. Thus, the studies described in this review use docking in the second stage, that is, after the experimental tests. However, there are other docking approaches that are used in a first stage, that is, before biological assessment to avoid spending on reagents and the irrational use of animal models. Therefore, instead of attempts to find potential compounds, other methodologies can be used to select the most promising compounds with the potential therapeutic effect even before biological assays.

With the advancement of more robust computational techniques, in silico studies guarantee greater reliability of results and rational drug planning.

Virtual screening is one of the methods that can be used in the investigation of compounds with antinociceptive activity. This method consists of screening chemical compound libraries using computational models or molecular docking in order to evaluate and/or select compounds with desired properties. Virtual screening is a fast and low-cost alternative for screening and selecting potential compounds for experimental evaluation (Alves et al., 2017). Docking is the main technique used in virtual screening based on structure. In this case, the molecules are coupled to the binding site and classified based on their predicted binding affinity or complementarity.

Pharmacophoric models based on structure are also a good alternative for the investigation of compounds with therapeutic potential. A pharmacophore model consists of a molecular recognition of a biological target shared by a group of compounds. Structure-based pharmacophores (SBPs) can work with either a free structure (apo) or a structure of the macromolecule-ligand complex (holo) (Pirhadi et al., 2013). These methods use the potential interactions observed between the ligand and the protein, while the SBP method, which aims to derive the pharmacophore from the free protein of the ligand, uses only information from the active site of the protein. This type of method also reduces costs and is considered a valuable tool for optimizing hits for leads, virtual screening, scaffolding jumping and design of drugs with multiple targets (Pirhadi et al., 2013).

Consensus docking uses various docking programs or various types of scoring functions to increase docking accuracy. The method helps to increase the classification power and, therefore, the hit rates, but combines information about the predicted connection modes instead of predicted connection affinities (Houston and Walkinshaw, 2013). Ao usar mais um programa de encaixe para visualizar uma pose de ligação, as poses podem atingir um índice de 82% (Houston and Walkinshaw, 2013).

### Different Molecular Docking Approaches Applied to Antinociceptive Studies

Most of the studies reported in this review describe the use of docking to investigate the mechanism of action, characterize interactions between targets and ligands, and assess antinociceptive activity at the molecular level. However, there are some Docking approaches that can be used to assess antinociceptive activity for different purposes.

An interesting study addressed by Poli et al. (2019), used virtual screening as a method to filter the compounds with the greatest potential to be tested experimentally. Considered one of the first examples of virtual screening studies focused on the identification of new peptide ligands as opioid modulators, the authors used parallel virtual screening from an internal library. The library containing 198,000 tetrapeptides was filtrated using a pharmacophore coupled to the X-ray structure of the µ-opioid receptor linked to the β-FNA morphine antagonist (PDB 4DKL code) using the LigandScout software. With this first filter, 28,070 compounds were selected and submitted to Docking using the software Glide. The software was able to select 146 compounds that were subjected to a second coupling using the Autodock Vina software. Vina was able to select 15 best compounds with probability of antinociceptive activity in opioid targets that were subjected to molecular dynamics studies to investigate the affinity of interactions in the presence of factors, such as solvent and ions. The three most promising peptide compounds were synthesized and subjected to biological evaluation. The results showed that peptide 1 showed selectivity for MOR and demonstrated an appreciable inverse agonist effect in MOR. The authors concluded that this peptide may represent a promisingly successful new compound to be used as a starting point for the optimization of structure-based ligands, with the aim of discovering potent opioid modulators.

Another study by Khanna et al. (2019) also used virtual screening to identify small molecules that disrupt the CaVα–CaVβ interaction. A commercially available library at ChemBridge was used to couple 50,000 small commercially available drug-like molecules. Of these compounds, 49 compounds were screened for their ability to inhibit calcium influx induced by depolarization in rat DRG neurons. These compounds were purchased, 13 were found to be insoluble or killed neurons, and 11 compounds inhibited the influx of Ca by 50%. The anchored compound, 2-(3,5-dimethylisoxazol-4-yl)-N-((4-((3-phenylpropyl)amino)quinazolin-2-yl)methyl)acetamide (IPPQ) was capable to interrupt the CaVα interaction CaVβ and considered as a non-opioid therapy for chronic pain.

An approach based on the design of compounds from docking and virtual screening was used by Lee et al. (2014). The researchers observed through bibliographic research that some compounds with a piperidine portion, such as haloperidol, penfluridol, pimozide, flunazirine, and TTA-P2, were well known as type T calcium channel inhibitors. Thus, inspired by these compounds and other types second generation, new compounds with greater potential for the treatment of neuropathic pain were designed. To predict a binding affinity of the projected compounds to the T-type calcium channel before synthesizing them, the researchers mapped the 3D ligand-based pharmacophore model generated by a common resource generation approach (HipHop) implemented in the CATALYST program (Doddareddy et al., 2007). The results showed that 4-phenyl compounds and 4-(3,4-dichloro) phenyl tetrahydropyridine compounds 7a and 7b showed bonds and interactions with the target similar to that of their tetrahydropyridine analogs 7. These compounds were evaluated for effect antinociceptive in rat models for neuropathic pain and were significantly effective in decreasing pain responses to mechanical mechanisms.

Daga et al. (2014), presented a combined method based on structure and ligand. They used a manual structure-based pharmacophore, which has the advantage of finding the binding conformation of the ligand in the bioactive state of the receptor. The structure of the nociceptin receptor (NOP), of the family of opioid receptors was used to fit with agonist ligands. Given the structure-activity relationships for known NOP ligands, the researchers developed a hybrid method that combines a structure-based and ligand-based approach, using the active state NOP receptor, as well as the pharmacophoric resources of ligands showed greater effectiveness than methods individual. The results showed that the NOP receptor binding affinity of a selected set of high-scoring hits resulted in the identification of several compounds with measurable binding affinity at the NOP receptor, one of which had a new chemotype for binding to the NOP receptor.

**Figure 2:**
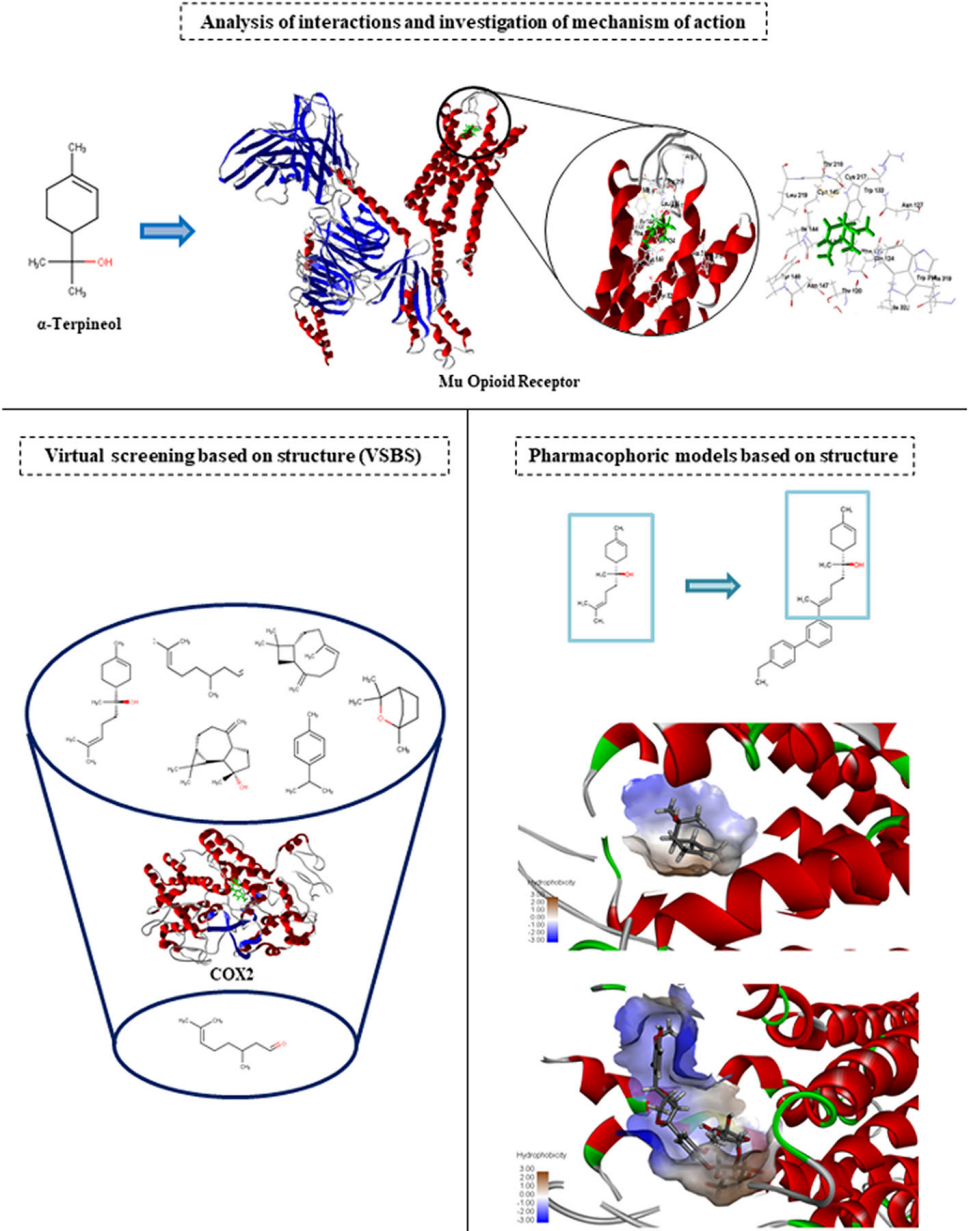
Different molecular docking approaches that can be applied in studies of antinociceptive activity. The interaction analysis allows to evaluate the main connections and interactions observed between compounds and targets before and after experimental tests. The virtual screening based on the structure consists of selecting selective compounds according to the binding affinity with the target protein. Pharmacophoric models based on structure consist of the molecular recognition of a target shared by a group of compounds with a similar structural base.

A summary of all docking approaches observed in this review is shown in **Figure 2**.

## Conclusion

The data presented in this review demonstrate the importance of studies with natural products, more specifically involving pharmacology with the help of bioinformatics/cheminformatics techniques, which is currently complementing and facilitating the discovery of new compounds, guiding and orienting studies towards specific molecular targets.

The most cited compounds in docking studies in this review are monoterpenes, especially α-Terpineol, (−)-α-bisabolol, Camphor, and p-Cymene. Many of the research addressed in this review used molecular docking to investigate the mechanism of action of various compounds and molecular dynamics to elucidate the stability of protein–ligand complexes in systems containing water, ions, temperature, and pressure. For new investigations, future perspectives include the advancement of more robust computational techniques and the increase *in silico* studies that complement the experimental studies, the tendency is to use computational resources in the first stage in the investigation of molecules with potential biological activity for certain diseases. Computational methods contribute to the selection of chemical structures with the highest probability of biological activity and the rationalization of these compounds. Several studies use QSAR (Quantitative Structure-Activity Relationship) methods to identify potential molecules with antinociceptive activity.

## Author Contributions

All authors contributed to the development of the article. DA, HA, DD, and HA held the discussion of the articles. RC, RB, and NB performed the methodology, bibliographic search, and selection of articles. MM and LS were in charge of generating the tables and discussing the content of chemoinformatics. MS and RA responsible for the general review of the content.

## Funding

This study was supported by funds from the Coordination for the Improvement of Higher Education Personnel (CAPES) and the National Council for Scientific and Technological Development (CNPq) and the Federal University of Paraíba (UFPB) for funding the article invoice.

## Conflict of Interest

The authors declare that the research was conducted in the absence of any commercial or financial relationships that could be construed as a potential conflict of interest.
